# Myocardial infarction due to malposition of ECMO cannula

**DOI:** 10.1007/s00134-012-2583-3

**Published:** 2012-04-26

**Authors:** Dinis Reis Miranda, Lara Dabiri Abkenari, Koen Nieman, Marcel Dijkshoorn, Eric Duckers, Diederik Gommers

**Affiliations:** 1 Department of Intensive Care, Erasmus Medical Centre, Gravendijkwal 230 H-619, 3015 CE Rotterdam, The Netherlands; 2 Department of Cardiology, Erasmus Medical Centre, Rotterdam, The Netherlands; 3 Department of Radiology, Erasmus Medical Centre, Rotterdam, The Netherlands

Dear Editor,

The most reported complications of veno-venous extracorporal membrane oxygenation (ECMO) are bleeding and infections [1]. Recently, a double lumen cannula was introduced, making introduction of two separated cannulas unnecessary. This single cannula is inserted via the right internal jugular vein and positioned with the tip resting in the inferior vena cava. Through ports in the superior and inferior caval veins, blood is returned directly into the right atrium. There have been reports describing ambulatory patients on veno-venous ECMO [2] with this type of cannula.

To date, only “minor” complications have been described such as displacement of the cannula tip from the inferior vena cava into the right atrium [3].

Here we describe a case of a displaced Avalon double lumen cannula, which caused compression of the right coronary artery, 14 days after initiation of ECMO.

A 61-year-old (height 1.80 m) man suffering from severe H1N1 pneumonitis for 4 days was referred to our tertiary center for ECMO. One day after admission at our center, a 27-Fr Avalon Elite bi-caval dual lumen catheter (Avalon Laboratories, Rancho Dominguez, CA, USA) was percutaneously inserted into the right jugular vein under fluoroscopy. With the catheter fully inserted, fluoroscopy showed that the tip just reached the inferior vena cava. The first X-ray confirmed this correct position.

On the third day, X-ray showed that the ECMO cannula projected into the right atrium. Many attempts to reposition the cannula under fluoroscopic control failed to obtain a stable position. The team therefore had to accept the position of the tip in the right atrium.

On the 14th ECMO day, cardiac markers were elevated. Echocardiography showed septal and inferior wall motion abnormalities and that the tip of the cannula was in the right atrium without crossing the tricuspid valve. On the suspicion of acute myocardial infarction, emergency coronary angiography was performed. Coronary angiography showed occlusion of the right coronary artery due to compression by the ECMO cannula (see electronic supplement: angiography in two directions). The suggested anatomic location of the compression of the right coronary artery is marked in Fig. 1. Withdrawal of the cannula resulted in a good coronary flow and after many attempts the tip of the cannula was placed in the inferior vena cava. On the 21st day, the patient was successfully weaned from the ECMO. On the 23rd day, the patient was transferred back to the referring hospital. Right ventricular function was normal and due to hypokinesia in the inferior segments; left ventricular function was moderate. Six months after hospital discharge, he had made an excellent recovery.

**Fig. 1:**
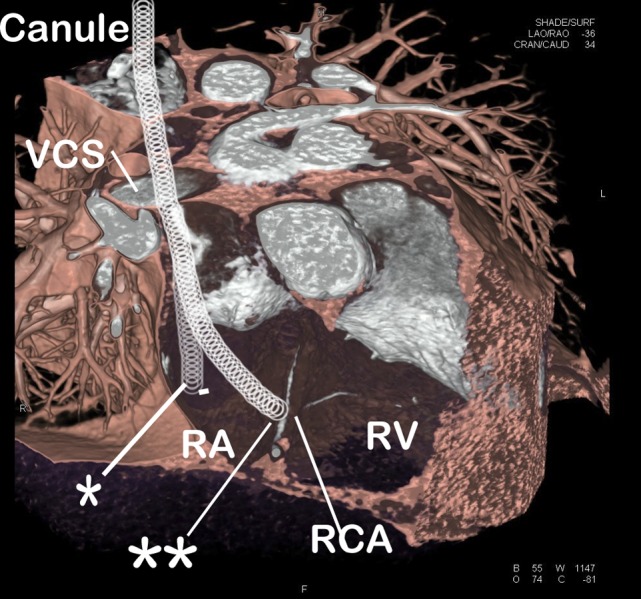
Three-dimensional computed tomography of the right atrium and right ventricle of the heart of a healthy subject (not the described patient). Note the right coronary artery (*RCA*) traveling on the epicardium between the right atrium and right ventricle. The correct cannula position is marked with an *asterisk*, with the tip in the inferior vena cava. Also, the suggested incorrect pathway of the cannula is marked with a *double asterisk* with the tip reaching the tricuspid annulus, compressing the RCA. *RA* right atrium, *RV* right ventricle, *VCS* vena cava superior

We conclude that when the cannula is short in relation to patient anatomy, misplacement of the cannula can easily occur and this could have serious complications.

## Electronic supplementary material

Below is the link to the electronic supplementary material.

Coronary angiogram showing compression of the right coronary artery by the ECMO catheter in the left anterior oblique view and perpendicularly, the right inferior oblique view (14 days after cannula insertion). (MP4 1,826 kb)
